# Detection of DDoS Vulnerability in Cloud Computing Using the Perplexed Bayes Classifier

**DOI:** 10.1155/2022/9151847

**Published:** 2022-07-19

**Authors:** Narendra Mishra, R. K. Singh, S. K. Yadav

**Affiliations:** ^1^Indira Gandhi Delhi Technical University for Women, Kashmere Gate, Delhi 110006, India; ^2^Department of Income Tax (Systems), Delhi, India

## Abstract

Cloud computing security has been a critical issue with its increase in demand. One of the most challenging problems in cloud computing is detecting distributed denial-of-service (DDoS) attacks. The attack detection framework for the DDoS attack is tricky because of its nonlinear nature of interruption activities, atypical system traffic behaviour, and many features in the problem space. As a result, creating defensive solutions against these attacks is critical for mainstream cloud computing adoption. In this novel research, by using performance parameters, perplexed-based classifiers with and without feature selection will be compared with the existing machine learning algorithms such as naïve Bayes and random forest to prove the efficacy of the perplexed-based classification algorithm. Comparing the performance parameters like accuracy, sensitivity, and specificity, the proposed algorithm has an accuracy of 99%, which is higher than the existing algorithms, proving that the proposed algorithm is highly efficient in detecting the DDoS attacks in cloud computing systems. To extend our research in the area of nature-inspired computing, we compared our perplexed Bayes classifier feature selection with nature-inspired feature selection like genetic algorithm (GA) and particle swarm optimization (PSO) and found that our classifier is highly efficient in comparison with GA and PSO and their accuracies are 2% and 8%, respectively, less than those of perplexed Bayes classifier.

## 1. Introduction

**Figure 1:**
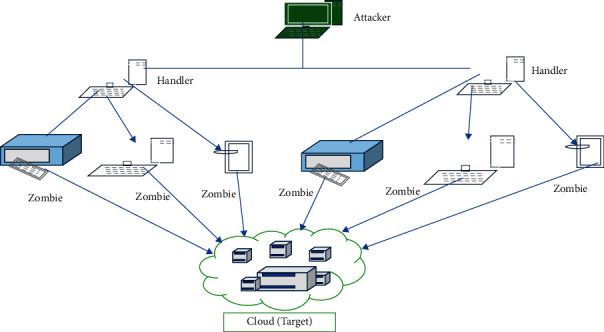
DDoS attack on Cloud [6].

It is feasible to provide a range of services through the Internet using cloud computing. Cloud computing provides an on-demand solution for various applications such as data storage, servers, databases, networking, and software. It provides convenient network-based access to shared pools of preconfigurable system resources and the ability to increase services on demand. The world is seeing unprecedented growth in cloud-enabled services. It is expanding exponentially to enjoy the advantages of improved efficiency, better scalability, load balancing, and faster deployments [1]. However, as cloud computing grew more prevalent, worries about data security, systems, and the development of cloud services became the most formidable job. In addition, several researchers found that all stakeholders of cloud computing users express that cloud service capability majorly affects cloud computing in mainstream adoption [2]. Identifying and exploiting vulnerabilities in cloud computing is challenging [3]. The DDoS assault is one of the most severe dangers in the era of cloud computing [4]. DDoS attacks are meant to knock a system/network down while also preventing its intended users from utilizing it. One of the most challenging difficulties in cloud computing is detecting distributed denial-of-service attacks. DDoS assaults, which overwhelm the target with excessive traffic, can potentially bring the system down [5]. DDoS attacks may occur both within and outside, disrupting cloud computing infrastructure.  Figure 1 explains the scenario of a DDoS attack performed on cloud computing where multiple systems (zombies) target the Cloud with a DDoS attack. The targeted network is then flooded with packets from different locations [7].

Cloud computing attacks include data threats, cloud service abuse, wrappers such as extensible markup language (XML) injection, man-in-the-cloud attacks, flooding assaults, and syn flood attacks [8]. A DDoS attack aims to overwhelm a system and prevent people from accessing services. These assaults are incredibly destructive to cloud computing platforms, preventing legitimate users from accessing cloud services [9].

The next stage deals with the DDoS assault. It keeps the different cloud computing services operational, which can only be accomplished by quickly identifying and mitigating cloud vulnerabilities in different ways. One of the most challenging difficulties in machine learning-based systems for identifying and mitigating DDoS cloud vulnerabilities is recognizing these assaults with high accuracy. Butt et al. [10] explained that the naïve Bayes classifier, random forest, artificial neural network (ANN), and decision tree are a few machine learning methods that the author has offered to solve cloud security challenges [11]. This algorithm uses a supervised and unsupervised approach by evaluating each technique's efficiency based on features and other parameters. However, the main drawback of this work is that it does not present as overwhelming to the naïve Bayes classifier.

Further, Eshtay et al., 2020 [12], Mishra and Singh [13], and Amine et al. [14] used particle swarm optimization (PSO) to handle the several issues related to cloud vulnerabilities. They attempted to propose a model/solution that increases the network lifetime and optimizes delivery. For this purpose, they have used an unsupervised learning approach for DDoS mitigation; however, due to its nonsupervised machine learning approach, the model needs to be enhanced for all possible DDoS attacks. This also has a limited source of vulnerability reporting and lower capabilities compared with naïve Bayes classifier, random forest, ANN, decision tree, etc. Similarly, Amjad et al. [15], to regulate and evaluate network traffic among virtual machines in a cloud environment, built an intrusion detection system employing two different methodologies in the form of the hybrid approach, namely, naïve Bayes classifier and random forest; however, due to the dependent variable feature, the above model does not cover all possible DDoS attacks. The identification and reporting time of vulnerabilities is lower compared with the other hybrid ML algorithm. In some cases, for example, Amjad et al. [16] used analysis of metrics and implementation procedures for evaluating the performance of existing techniques and presented their observations accordingly.

Similarly, Singh et al. [17] obtained the implementation results by engaging individual classifiers with the combined result of all the four classifiers with intrusion detection models. Implementation results demonstrate the proposed model's ability with an accuracy of 97.24%; however, the accuracy was very low, and the proposed model is less effective than the other existing model in identifying DDoS vulnerabilities. Mahmood et al. [18] introduced the hidden naive Bayes (HNB) classifier to manage DDoS attacks by relaxing the conditional independence requirement of cloud computing systems. According to their findings and the HNB classifier, detecting DDoS vulnerabilities is more than 90% accurate; however, the main limitation of their study is that they only chose 10–12 characteristics, which leads to less efficient DDoS vulnerability detection. During the initial research studies, several researchers identified the extensive use of supervised machine learning (primarily the naïve Bayes classifier) to detect and mitigate DDoS attacks; however, due to the limitation of the independent variable in the naïve Bayes classifier, accuracy is always a big concern for all [19]. To address the above problem effectively, the suggested perplexed-based classifier with the feature for identifying DDoS vulnerabilities of cloud computing would be a new avenue for researchers to improve cloud computing efficiency. The below-mentioned steps are the essential contribution and further describe the key findings of this paper.

- The proposed perplexed Bayes classifier model for DDoS attacks in cloud computing uses the NSL-KDD+ data set to train on 70% data set and the remaining (30 per cent data set) for its testing
- A feature selection approach based on correlation value is utilized with a perplexed Bayes classifier to investigate the increased accuracy of detecting DDoS attacks in cloud computing on the same data set
- To investigate the performance parameters, compare the above two suggested methodologies with naïve Bayes and random forest algorithms

This research presents a unique technique called perplexed Bayes classifiers identifying DDoS attacks in cloud computing services, in which the data set comprised several DDoS attacks and their associated features. The significant features for detecting DDoS attacks in cloud computing will be chosen based on correlation, and the available data set features will be trained into the proposed algorithm. To demonstrate the usefulness of the new approach, performance measurements have been used to compare it to current algorithms such as perplexed-based classifiers with and without feature selection, naïve Bayes classifiers, and random forest techniques and further with nature-inspired computing algorithm like GA and PSO. This suggested algorithm will work for all DDoS attacks with characteristics independent of one another. Although this study focuses purely on DDoS attacks, this approach may be used for any attack in cloud computing when the characteristics are not interdependent.

## 2. Literature Survey

Several papers on DDoS defence solutions in cloud computing are closely linked to our study in the following literature. On the one hand, only a few authors concentrated on DDoS attack detection and mitigation, and, on the other hand, some authors attempted to review the processes for detecting and mitigating DDoS attacks. At the same time, our work has done a more thorough investigation with more technical details than these existing evaluations. We have compiled a list of research gaps for this study and tried to find out that these review papers listed in Table 1 do not address the genuine concern for detection and mitigation of DDoS attacks in cloud computing.

## 3. Methodology

This research aims to implement machine learning techniques, i.e., perplexed-based classifier, to identify and mitigate DDoS attacks over a cloud environment. The features are extracted with the priority of correlation value. These extracted features will be trained to the proposed algorithm for detecting DDoS attacks. To implement this, we have used Python. The chosen data set, features of the data, and all the preprocessing and analysis steps to be implemented are described below. The feature selection (FS) approach determines what data will be extracted from the available network traffic flow for examination by the IDS model [31]. The purpose is to enhance the performance of the IDS by creating an optimal set of features. The supervised, unsupervised, and semisupervised feature selection methods can act as a very efficient way to reduce data redundancy and improve performance.

### 3.1. Data Set

The NSL-KDD data set (https://www.kaggle.com/datasets/towhidultonmoy/kddcup98-dataset, https://www.kaggle.com/code/farelarden/nsl-kdd-randomforest-w-optuna, and https://www.unb.ca/cic/datasets/nsl.html) is the revised, updated, and cleaned version of KDD-99 data set of the University of New Brunswick. This has been used in our research paper [32].This database contains a standard set of data, including the intrusions simulated in a network environment. Further, the data set was generated by capturing raw TCP/IP dump data by simulating a LAN (local area network). The data set consists of 43 features (listed in Table 2), out of which 41 features dealt with traffic input features and the remaining two features represents the label (whether there is attack or normal (no attack)) and score (severity of the attack). The size of the data set contains 22,544 rows and 43 features, and it covers all DDoS attacks and is used by several researchers for the machine leaning algorithm. The attack classes of this data set cover the following [35]:

1. Distributed denial of services (DDOS)
2. Probe (PR)
3. Root to local (R2L)
4. User to root (U2R)

The brief schema of the data set is listed in Table 3.

### 3.2. Data Preprocessing

**Figure 2:**
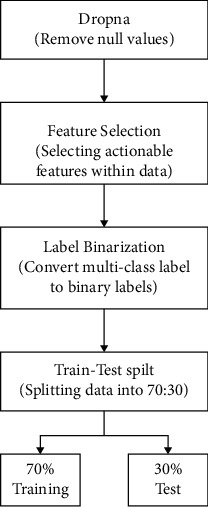
Flowchart of data preprocessing.

Data preprocessing can be referred to as a step within data mining used to perform a data analysis process that takes raw data as input and transform it into the desired format [36]. This is an initial and essential footstep in the data mining process. Here, the data chosen will undergo the listed preprocessing steps proposed in Figure 2 to fit the proposed model.

### 3.3. Elimination of Null Values

Null values interpret all the necessary actions of analysis like plotting and model fitting. If there are any null values in the data, they need to be removed by using dropna() since they mislead the findings. The overall size of the data before dropna() is 22544*∗*43 and after dropna data size is 22536*∗*43 along with 8 missed values.

### 3.4. Correlation

Features are selected based on the correlation value with the target variable presented in Table 4. Correlation generally assesses the magnitude and direction of a linear relationship between two or more variables. If the correlation value is 1, the variables are strongly correlated, and if the value is −1, variables are negatively correlated. If the correlation value is 0, the variables are not correlated. Hence, to find the actionable features, the feature should strongly correlate with the target variable. Once the features are selected, a sample of 20 features will be taken from the extracted features to train the model.

### 3.5. Label Binarization

This is for converting the multiclass labels to binary labels, making the data easily accessible and efficient in training the model. The train-test split approach takes a data set and divides it into two divisions. The training data set is the starting point for fitting the model. The data set involvement section is provided to the archetypal, who further marks assumptions and relates those to the predicted values. The second select group is not used to train a model; instead, the data set's feedback aspect is provided to the framework, further trying to predict and equate those to the estimated parameters. The test data set is presented as the second data set. The whole data set is partitioned into a 70 : 30 ratio. The training accounts for 70% of the data, and data testing accounts for 30%.

### 3.6. Data Analysis

The data set features are initially correlated to extract some actionable features from the data, and these features will be trained into the perplexed-based classification algorithm. Regardless of the type of DDoS attack, all the attacks will be labelled 1, and the normal connection will be labelled 0, making the data set binary form for binary classification.

### 3.7. Correlation

A statistical term correlation is defined as a linear link between two variables. It is a distinctive method of discussing fundamental relationships, deprived of overtly articulating a cause-and-effect relationship. This correlation technique will show us how the data features strongly correlate to the target variable. The highly correlated features with the target are selected, which holds maximum variation. Hence, these features are highly recommended for better accuracy. This is supported by the result of a perplexed-based classifier with feature selection and a perplexed-based classifier without feature selection.

## 4. Results and Discussion

### 4.1. Perplexed-Based Classification Implementation in the Cloud

The perplexed Bayes classifier is a mathematically superior variant of the naïve Bayesian classification technique. It is a classifier that works similarly to the naïve Bayes classifier; however, given the absence of the postulate of “conditional class independence,” it is termed the perplexed Bayes classifier (the geometric mean) because it uses the reciprocal of perplexity to aggregate the probability of selected characteristics into a single value [37]. Because of the nonlinearity of the data, the proposed perplexed algorithm handles the data as there is no interdependence within the system traffic data.

Probabilistic classifiers choose the most likely class based on the features of the data item being categorized, as shown in equation (1).

(1) $$\begin{array}{c} arg\underset{c}{\max}P\left(C|A\right). \end{array}$$

Bayesian classifiers convert *P*(*A|C*) to *P*(*C|A*), as shown in equation (1).

(2) $$\begin{array}{c} P\left(C|A\right)=\frac{P\left(A|C\right)\times \;P\left(C\right)}{P\left(A\right)}. \end{array}$$

In addition, naïve Bayes classifiers assume that the characteristics f1, f2, f3, and so on are independent of one another, conditional on class C, resulting in equation (3).

(3) $$\begin{array}{c} P\left(A|C\right)=\prod_{i}P\left(a_{i}|C\right). \end{array}$$

Equation (4) is obtained by substituting equation (3) into equation (2).

(4) $$\begin{array}{c} P\left(C|A\right)\;=\frac{\left(\prod_{i}P\left(a_{i}C\right)\right)\times P\left(C\right)\;}{P\left(A\right)}. \end{array}$$

Equation (4) produces a lot of extreme posterior probability values. Naïve Bayes classifiers might be more effective for NLP if their posterior probability estimations were improved.

Equation (5) shows how to determine the perplexity PP(*p*_1_, *p*_2_,…*p*_*n*_) of a collection of probabilities {*p*_1_, *p*_2_,…, *p*_*n*_}:

(5) $$\begin{array}{c} \text{PP}=\frac{1}{{\left(p1\times p2\times \ldots \times pna_{i}\right)}^{1/na_{i}}}. \end{array}$$

In the perplexed Bayes classifier, we use the geometric mean to integrate the class conditional feature probabilities, as indicated in equation (6).

(6) $$\begin{array}{c} P\left(A|C\right)={\left(\prod_{1\leq i\leq na_{i}}P\left(a_{i}|C\right)\right)}^{1/na_{i}}. \end{array}$$

As a result, equation (8) may be represented as the posterior probability equation, whereas *n* is the no. of features and N is the normalizer, where the posterior probability is presented in equation (7):

(7) $$\begin{array}{c} \text{Posterior probability}=\text{prior probability}+\text{new evidence.} \end{array}$$

(8) $$\begin{array}{c} P\left(C|A\right)=\frac{\prod_{i}P{\left(a_{i}|C\right)}^{1/na_{i}}\times P\left(C\right)\;}{N}. \end{array}$$

All the above equations from (1) to (8) derived by Haq et al. [38] and Carlos et al. [39]. The given performance metrics indicate the effectiveness of the DDoS attack detection.

### 4.2. Performance Metrics

The confusion matrix's performance characteristics, such as accuracy, sensitivity, and specificity, assess the suggested algorithm's performance. Dhingra and Yadav [40] presented and discussed the following equations (9) to (11) in their research work.

#### 4.2.1. Accuracy

Accuracy is defined as the fraction of properly recognized subjects to the total number of subjects. The expression for accuracy is given in equation (9):

(9) $$\begin{array}{c} \text{Accuracy}=\frac{\text{TP}+\text{TN}}{\text{TP}+\text{TN}+\text{FP}+\text{FN}}. \end{array}$$

#### 4.2.2. Sensitivity

Recall, also known as sensitivity, is the proportion of correctly positive labels recognized by our classifier. The expression for sensitivity is given in equation (10):

(10) $$\begin{array}{c} \text{Sensitivity}=\frac{\text{TP}}{\text{TP}+\text{FN}}. \end{array}$$

#### 4.2.3. Specificity

**Figure 3:**
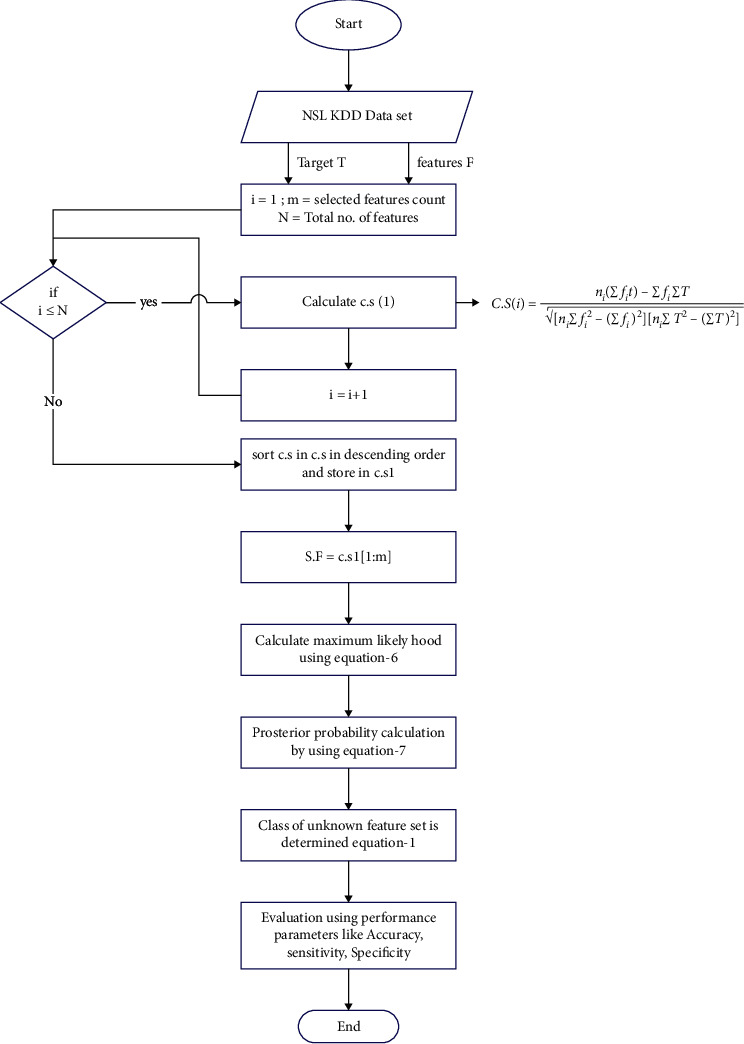
Flowchart of the proposed methodology.Here, cs = correlation score, Ni = number of elements in a feature, fi = ith feature, and T = target.

The system has appropriately classified the negative as specificity. The expression for specificity is given in equation (11):

(11) $$\begin{array}{c} \text{Specificity}=\frac{\text{TN}}{\text{TN}+\text{FP}}, \end{array}$$

where TP = true positive, FP = false positive, TN = true negative, and FN = false negative. The above proposed algorithm is visualized in the flowchart presented in Figure 3.

Further, the correlation technique has been used to identify the actionable features, and comparisons of the proposed algorithm with other algorithms following performance parameters are displayed.

**Figure 4:**
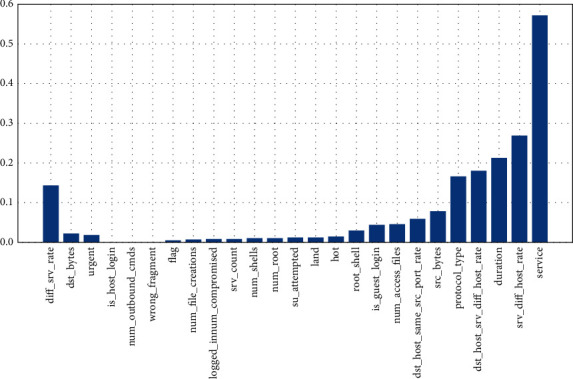
Correlation of features with target variables.

Figure 4 depicts the correlation between the features and the target variable. It is observable that service is the feature that is highly correlated with the target variable.

**Figure 5:**
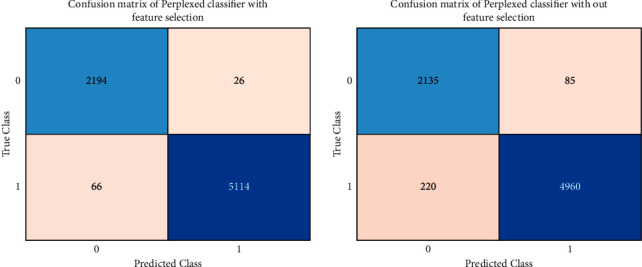
Confusion matrix of the perplexed classifier (a) with and (b) without feature selection.

The confusion matrix of the perplexed classifier with feature selection is displayed in Figure 5. The two classes are defined in the matrix, where 0 is normal and 1 is the attack. The proposed algorithm had accurately predicted the regular attacks 2194 times and malicious attacks 5114 times. There have also been misinterpretations of the proposed model, with one class being misinterpreted as another. At the same time, the perplexed classifier's confusion matrix without feature selection is shown above. The model had accurately predicted the regular attacks 2136 times and the malicious attacks 4960 times of the data. There have also been misinterpretations of the model, with one class being misinterpreted as another.

**Figure 6:**
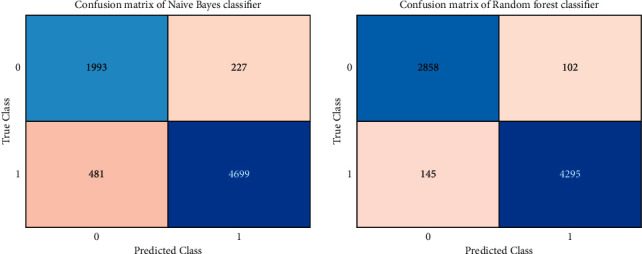
Confusion matrix of naïve Bayes classifier (a) and random forest classifier (b).

The confusion matrix of the naïve Bayes classifier is seen in Figure 6. The model correctly predicted the data for regular attacks 1993 times and malicious attacks 4699 times. Misinterpretations of the model have also occurred, with one class being misinterpreted as another. At the same time, the random forest classifier's confusion matrix may be seen above. The model accurately predicted the regular attacks 2858 times and abnormal attacks 4295 times within the data. There are also misinterpretations where the model had inaccurately predicted one class with another.

**Figure 7:**
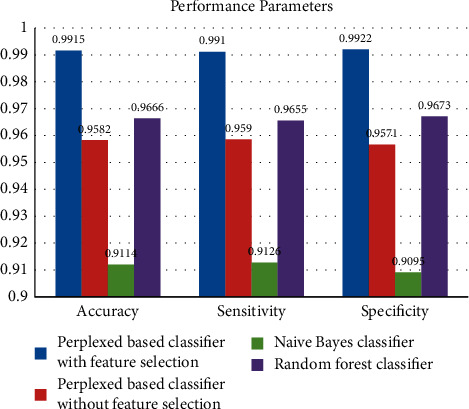
Performance parameters of the algorithms (PBC/F, PBC/WF, NBC, and RF).

The suggested algorithm's performance parameters, such as accuracy, sensitivity, and specificity, and that of the other two existing algorithms are displayed in Figure 7. The accuracy of the proposed algorithm is 0.9915, the accuracy of the random forest classifier is 0.9666, the classifier's accuracy without feature selection is 0.9582, and the naïve Bayes accuracy is 0.9114. Hence, the accuracy of the proposed algorithm is high at approximately 3% with random forest classifier and approximately 4% and 8%, respectively, with classifier without feature selection and naïve Bayes classifier. The specificity of the proposed algorithm is 0.9922, and without feature, the selection is 0.9571, that of the naïve Bayes classification is 0.9095, and that of the random forest classifier is 0.9673. Hence, the specificity of the proposed algorithm is higher by approximately 3% with random forest and 4% and 9%, respectively, with classifier without feature selection and naïve Bayes, and their specificity are 0.9673, 0 0.9571, and 0.9095, respectively. The algorithm's sensitivity is 0.991, without feature selection is 0.959, that of naïve Bayes is 0.912, and that of the random forest is 0.9655. It is observable that the proposed algorithm is more effective in performance parameters than the other two existing algorithms. The values of the metrics of the three algorithms are tabulated in Table 5.

**Figure 8:**
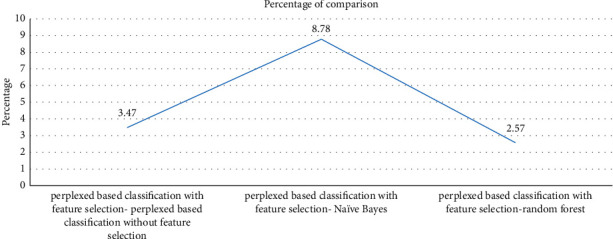
Percentage of comparison with the proposed algorithm.

In Figure 8, presented as a graph, the percentage of existing algorithms such as perplexed-based classifier without feature selection, naïve Bayes, and random forest algorithm is compared with the proposed algorithm, perplexed-based classification with feature selection. When comparing the accuracy of perplexed-based classification with feature selection with that of perplexed-based classification without feature selection, it is found that the accuracy of perplexed-based classification with feature selection improved by 3.47%. Compared with naïve Bayes, the accuracy of perplexed-based classification with feature selection improved by 8.78%. The perplexed-based classification with feature selection improved by 2.57% compared with the random forest method. As a result, when compared with existing methods, the suggested approach has higher accuracy and efficiency in identifying DDoS attacks in cloud computing.

### 4.3. Nature-Inspired Feature Selection versus Perplexed Bayes Classifier with Feature Selection

#### 4.3.1. Nature-Inspired Computing

Nature-inspired computing (NIC) is based on natural phenomena and behaviour to solve complex problems in various environmental circumstances and decision-making ability [37]. This has covered the algorithms such as GA, neural networks, and PSO. The algorithm that nature-inspired computing uses is primarily known as nature-inspired algorithm. Nature-inspired algorithms are step-by-step solutions, methodologies, and approaches to any computing problems that emerge from natural processes. Some famous examples of nature-inspired optimization algorithms include GA, PSO, and ant colony optimization, which are frequently used for vulnerability identification and mitigation in cloud computing.

#### 4.3.2. Feature Selection

In feature selection, the number of input variables has reduced to develop a predictive model, which reduces the computational cost of modelling and improves the model's performance. Within the available features, some actionable features are selected based on their priority score by correlation. The priority is estimated by finding the correlation of the feature to the target variable. The most correlated feature will be considered essential, while the less correlated features will be considered unessential.

This correlation-based selection is compared with nature-inspired feature selection like GA and PSO. The accuracy of the feature selection algorithm is as follows.

**Figure 9:**
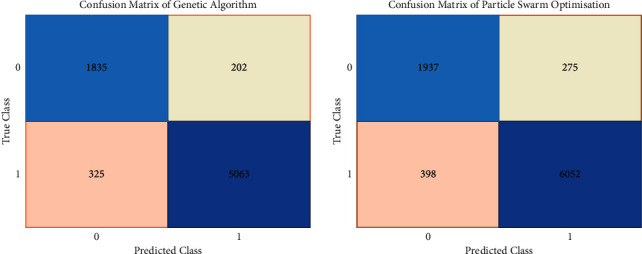
Confusion matrix of GA classifier (a) and PSO (b).

The confusion matrix of the GA is presented in Figure 9. The model correctly predicted the data for regular attacks 1993 times and malicious attacks 4699 times. Misinterpretations of the model have also occurred, with one class being misinterpreted as another. At the same time, the PSO confusion matrix may be seen as in Figure 9. The model accurately predicted the regular attacks 2858 times and abnormal attacks 4295 times within the data. There are also misinterpretations where the model had inaccurately predicted one class with another.

Table 6 depicts the accuracy comparison of feature selection with correlation, GA, and PSO. The accuracy of GA is 0.9744, i.e., 97%; the accuracy of PSO is 0.9119, i.e., 91%; and the accuracy of correlation is 0.9915, which is 99%. Similarly, the sensitivity of GA is 0.9655, i.e., 96%; the sensitivity of PSO is 0.9555, i.e., 95%; and the sensitivity of correlation is 0.9910, i.e., 99%. The specificity of the GA is 0.9673, i.e., 96%; the specificity of PSO is 0.9766, i.e., 97%; and the specificity of the correlation is 0.9922, i.e., 99%.

**Figure 10:**
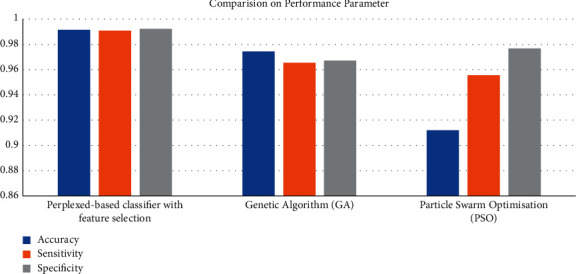
Performance comparison of feature selection and nature-inspired feature selection.

Hence, the correlation algorithm is highly efficient compared with the optimization algorithms GA and PSO on performance parameters and overall approximately 2% and 8%, respectively, less than the correlation, which can also be seen in Figure 10. Hence, the proposed algorithm benefits feature selection when compared with nature-inspired algorithms.

## 5. Conclusions and Future Work

Machine learning is used to find and choose data to identify DDoS assaults on cloud computing platforms. A novel approach, perplexed-based classification with feature selection, is presented to extract actionable characteristics and differentiate attacks from data. The data set containing characteristics linked to the assault is selected. The actionable features are extracted from the features by correlating them to the target variable. A sample of 20 features is selected and trained to the proposed model to detect DDoS attacks within the extracted features. To illustrate its efficiency, the suggested method is compared with others using performance measures. Service is substantially connected with the goal variable, per the correlation. The proposed algorithm is compared with others following performance parameters to prove its efficiency. It is observable from the correlation that the feature “Service” is highly correlated with the target variable. Hence, service features need to be more focused on detecting DDoS attacks. Compared with performance parameters like accuracy, sensitivity, and specificity, the proposed algorithm has an accuracy of 99%, which is higher than the existing algorithms, proving that the proposed algorithm is highly efficient in detecting the DDoS attacks in cloud computing systems. This suggested algorithm will work for all attacks with characteristics independent of one another. Although this study focuses purely on DDoS attacks, this approach may be used for any attack in cloud computing when the characteristics are not interdependent. In addition to that, when it was compared with the nature-inspired-based feature selection like (GA) and (PSO), our proposed perplexed Bayes classifier feature selection is highly efficient in comparison with the Nature Inspired Computing algorithm as optimization algorithms like GA and PSO accuracies and are lesser approximately 5% and 2%, respectively, than the perplexed Bayes classifier.

However, we can consider the collaborative and distributed detection of DDoS vulnerabilities in future work, emphasizing the emerging trend of distributed cloud computing and machine learning techniques for identification and mitigation. With the unique nature of DDoS attacks, approaches that combine collaboration, distribution, and even mobility with machine learning and other techniques, we may develop some more classifiers that provide better performance and cover both supervised and unsupervised machine learning approaches. In addition to that, to enhance cloud computing attack detection in more automated way, future research may use an optimized approach to evaluate IP source address, acknowledgement, reset, finished, TCP/IP, ICMP segments, and ports in more effective way, as DDOS attacks influence these parameters majorly.

## Figures and Tables

**Table 1:** Comparison of various DDoS attack studies.

Author	Year	Description	Remarks
Berguig et al. [20]	2018	1. The author of this work chose the KDD-CUP-99 data set.	The mobile-based strategies have been focused on resisting the DDoS attacks; however, the web-based strategies that were not covered could have also been covered.
		2. The authors provide the most extensively used mobile agent-based DDoS flooding assault defence tactics, a unique denial-of-service filter system based on mobile agents and naïve Bayes filters.	

Nandi et al. [21]	2020	1. The authors of this work had chosen the essential characteristics from the NSL-KDD data set.	The authors did not attempt to create a DDoS detector with actual traffic in a real-world cloud system.
		2. The paper employed a hybrid technique in which a five-feature selection algorithm chooses and ranks the top most significant characteristics from the whole feature set.	

Kim et al. [22]	2020	1. This study developed an intrusion model. Deep learning identifies DDoS attacks using the KDD-CUP 1999 data set and CSE-CIC-IDS 2018.	The data sets chosen for implementation also contain other classes of attacks. Hence, multiclass classification is not implemented in the current research.
		2. The implementation considered four attack types: DDoS, U2R, R2L, and probing.	
		3. The machine learning technique CNN, which is further compared with RNN, has been used.	

Cil et al. [23]	2021	1. This research uses deep neural network (DNN) to detect DDoS attacks on packet samples captured from network traffic.	It can create data sets like the CIC DDoS 2019 data set. It may be able to classify real-time DDoS attacks. By utilizing the data set, DNN and deep learning replicates will be built.
		2. The implementation is carried out with CIC DDoS 2019 data set to contain current DDoS attacks.	
		2. Feature extraction, the classification process of the structure, is done to train the data set to the model.	

Rangapur et al. [24]	2022	1. In this research, DDoS attacks are detected by using neural networks.	The data set consisting of different classes could be taken for implementation to improve the model's efficacy.
		2. The main focus is to flag malicious and legitimate data flow and to prevent network performance degradation.	

Saroha and Singh [25]	2019	1. The paper provides a qualitative analysis of all possible cloud vulnerabilities on each service model.	This study does not look at integrating into a cloud environment. No implementation was done for robust cloud systems. Also, the works do not use an ML algorithm.
		2. They have also proposed a countermeasure to enhance the security in cloud computing.	
		3. Characterization of vulnerabilities has been presented.	

Goel et al. [26]	2014	1. The author discussed cloud security vulnerabilities, dangers posed by a distributed denial-of-service (DDOS) assault on cloud computing infrastructure, and methods and tactics for detecting and preventing such attacks.	The paper had concentrated more on detection but not on mitigation.
		2. The author focused on and suggested an integrated and comprehensive model based on an intrusion detection system that addressed both internal misuse and external intrusion and that will detect or report the alert and vigorously challenge the attacks, reducing the overall risk of DDoS attacks.	

Deshmukh et al. [27]	2015	1. The author discussed DDoS attacks, their impact on cloud computing, and the factors to consider when picking DDoS security systems.	VM attacks may degrade cloud performance, result in financial losses, and impact other servers in the same cloud architecture.
		2. The author gave a quick overview of DDoS assaults, followed by a taxonomy of attacks, kinds of attacks, and several countermeasures to reduce DDoS attacks.	

Masdari and Jalali [28]	2016	1. The author has conducted an in-depth examination of the numerous forms of DDoS attacks suggested for the cloud computing environment, classifying them according to the cloud components or services they target.	There is no distinction between flash crowds and DoS assaults in clouds with dynamic material.
		2. It also included a thorough examination of the vulnerabilities used in various DoS assaults and an examination of the state-of-the-art solutions published in the literature for preventing, detecting, and dealing with each kind of DoS attacks in the Cloud.	

Oberoi [29]	2017	1. The author investigated various security attacks (in general) concerning clouds.	This study does not offer a system to identify harmful insider assaults in cloud-based settings with accuracy and timeliness.
		2. Insider threat assaults should not be taken lightly, according to the available literature (research papers, reports, etc.).	
		3. These assaults should not be taken lightly. The companies explicitly define the many categories of people capable of launching insider attacks and the dangers they face.	

JeyaJothi et al. [30]	2022	1. In this study, to achieve higher quality classification, the fast correlation-based feature selection (FCBF) method was used for data preprocessing and further to remove irrelevant and redundant features of the data.	This has a limitation as it selects some limited features of the data set. The data pre-preprocessing could be done in a better way. Any new classifier may be used to achieve the best result.
		2. SVM classification has been done using a linear approach.	
		3. Its limitation to dependent feature, which carries investigations, carried out feature extraction and its optimization techniques for OSA detection.	

**Table 2:** Features of the data used in the data set [32–34].

List of features of the data			
Duration	logged_in	Count	dst_host_same_srv_rate
protocol_type	num_compromised	srv_count	dst_host_diff_srv_rate
Service	root_shell	serror_rate	dst_host_same_src_port_rate
Flag	su_attempted	srv_serror_rate	dst_host_srv_diff_host_rate
src_bytes	num_root	rerror_rate	dst_host_serror_rate
dst_bytes	num_file_creations	srv_rerror_rate	dst_host_srv_serror_rate
Land	num_shells	same_srv_rate	dst_host_rerror_rate
wrong_fragment	num_access_files	diff_srv_rate	dst_host_srv_rerror_rate
Urgent	num_outbound_cmds	srv_diff_host_rate	Label
Hot	is_host_login	dst_host_count	Severity
num_failed_logins	is_guest_login	dst_host_srv_count	

**Table 3:** Details of NSL-KDD data set [32–34].

Since	Data set	Category	IP address	Redundancy	Availability	Features	Last updated
1999	NSL-KDD	Real	Mapped	No	Yes	43	04-06-2022

**Table 4:** Target features [32–34].

Normal and abnormal attacks				
Normal	Spy	Nmap	Smurf	Neptune
	Teardrop	Back	Imap	Multihop
	Warezclient	Rootkit	guess_passwd	Land
	Loadmodule	Satan	ftp_write	Ipsweep
			Buffer overflow	Warez master

**Table 5:** Performance comparison of the algorithms (PBC/F, PBC/WF, NBC, and RF).

Algorithm	Accuracy	Sensitivity	Specificity
Perplexed-based classifier with feature selection	0.9915	0.9910	0.9922
Perplexed-based classifier without feature selection	0.9582	0.9590	0.9571
Naïve Bayes classifier	0.9114	0.9126	0.9095
Random forest classifier	0.9666	0.9655	0.9673

**Table 6:** Performance comparison of the algorithms (PBC/F, GA and PSO).

Algorithm	Accuracy	Sensitivity	Specificity
Perplexed-based classifier with feature selection	0.9915	0.9910	0.9922
Genetic algorithm (GA)	0.9744	0.9655	0.9673
Particle swarm optimization (PSO)	0.9119	0.9555	0.9766

## Data Availability

The data can be accessed at https://www.unb.ca/cic/datasets/nsl.html, https://www.kaggle.com/datasets/towhidultonmoy/kddcup98-dataset, and https://www.kaggle.com/code/farelarden/nsl-kdd-randomforest-w-optuna.
